# Saporin Conjugated Monoclonal Antibody to the Transcobalamin Receptor TCblR/*CD*320 Is Effective in Targeting and Destroying Cancer Cells

**DOI:** 10.4236/jct.2013.46122

**Published:** 2013-08-01

**Authors:** Edward V. Quadros, Yasumi Nakayama, Jeffrey M. Sequeira

**Affiliations:** 1 Department of Medicine, SUNY-Downstate Medical Center, Brooklyn, USA; 2 Department Cell Biology, SUNY-Downstate Medical Center, Brooklyn, USA

**Keywords:** Transcobalamin Receptor, *CD*320 Gene, Cobalamin, Cancer, Toxin

## Abstract

Cobalamin uptake into cells is mediated by the *CD*320 receptor for transcobalamin-bound cobalamin. Optimum receptor expression is associated with proliferating cells and therefore, in many cancers this receptor expression is up regulated. Delivering drugs or toxins via this receptor provides increased targeting to cancer cells while minimizing toxicity to the normal tissues. Saporin conjugated monoclonal antibodies to the extracellular domain of TCblR were effectively internalized to deliver a toxic dose of Saporin to some cancer cell lines propagating in culture. Antibody concentration of 2.5 nM was effective in producing optimum inhibition of cell proliferation. The cytotoxic effect of mAb-Saporin appears to be dictated primarily by the level of receptor expression and therefore normal primary cells expressing low levels of *CD*320 were spared while tumor cell lines with higher *CD*320 expression were destroyed. Targeting the pathway for cellular uptake of vitamin B_12_ via the *CD*320 receptor with toxin-antibody conjugates appears to be a viable treatment strategy for certain cancers that over expresses this receptor.

## 1. Introduction

The transcobalamin receptor TCblR/*CD*320 [1] is ubiquitously expressed in most cell types and mediates uptake of transcobalamin (TC), a plasma protein saturated with cobalamin (Cbl, B_12_). Cbl in blood is bound to two plasma proteins; haptocorrin (HC) which carries ~70% of the vitamin and TC which is ~30% saturated with Cbl. The latter, a nonglycosylated protein secreted by vascular endothelial cells [2] has a relatively short half life and plays the all important role of transporting the newly absorbed Cbl in the distal ileum to all tissues where it binds to the receptor on the cell surface with high affinity [3]. TC-Cbl is internalized by receptor-mediated endocytosis of the ligand [4,5]. It is generally accepted that the TC is degraded in the lysosome and the Cbl is released [6]. Even though published data supports recycling of the receptor [7,8], unequivocal evidence in support of this is lacking. TCblR expression appears to be cell cycle associated with highest expression in actively proliferating cells and is substantially down regulated in quiescent cells [7–9]. This differential expression of the receptor serves to provide optimum delivery of the vitamin to cells during the early phase of DNA synthesis. This process ensures adequate functioning of Cbl dependent enzymes, especially the methionine synthase that is essential for recycling of methyl folate to generate folates needed for purine and pyrimidine biosynthesis. The more proliferative a cell, higher the need for folates and Cbl and this need for Cbl is met by the increased expression of TCblR in cancer cells that may have inherently lost the ability to stop dividing and differentiate. Selective targeting of cancer cells for destruction by delivering drugs and toxins preferentially to these cells has been the ultimate objective of cancer therapy. The search for tumor specific markers and the strategies to utilize these in cancer therapy have been pursued for decades with mixed results. Success in this approach has been limiting and can be attributed to multiple factors that include the lack of specificity of the target antigen, cellular events that can alter this targeting and to the complex and diverse nature of cancer itself. TCblR is structurally related to the LDL receptor family with two LDLR type A domains separated by a CUB like domain and contains a single transmembrane region followed by a short cytoplasmic tail [1]. We have generated monoclonal antibodies to the recombinant extracellular 200aa domain of TCblR and have defined the epitope specificity of these antibodies [10]. We have previously demonstrated the utility of anti TCblR antibody to deliver secondary antibody conjugated to Saporin [11], an inhibitor of ribosomal assembly [12] to cancer cells propagating in culture [13,14]. The present study extends this preliminary observation by demonstrating enhanced targeting and destruction of cancer cells by Saporin conjugated directly to monoclonal antibodies to the extracellular domain of TCblR/*CD*320.

## 2. Methods

All tumor cell lines obtained from ATCC were cultured to passage 2 to 4 and frozen for use in this study. MCH 064, MCH 065 and RF peripheral skin fibroblast cultures in passage 9 – 12 were from The Repository for Mutant Human Cell Strains, Montreal Children’s Hospital, Canada. Fresh human bone marrow mononuclear cells were obtained from Lonza, Walkersville, MD. Human chorionic trophoblasts, ED cells were obtained as previously described [15]. Cells were cultured in Dulbecco’s modified Eagle’s minimal essential medium (DMEM) with 10% fetal bovine serum (FBS) and antibiotics. Monoclonal antibodies generated to the extracellular domain of TCblR [10] were purified by affinity chromatography on a protein G agarose column and used for covalent conjugation of Saporin. The coupling of Saporin and purification of monoconjugate of the antibody was done at Advanced Targeting Systems, San Diego, CA. The antibody-Saporin conjugates were stored in aliquots at −20°C and used in the present study. All data reported represent mean results of duplicate analyses.

### Determination of optimum concentration of mAb-Saporin conjugate

HEK293 cells (2 × 10^3^ in 100 ul complete DMEM) were seeded in 96 well culture plates with 0.156 – 5 nM mAb-Saporin for 72 hours and viable cells were quantified using the CellTiter96 Aqueous One solution cell proliferation assay (Promega). Optical density at 495nm was read in a microplate reader (Bio-Rad). Statistical analysis was done using GraphPad software Prism 3.03.

### Effect of Cell seeding density on efficacy of mAb-Saporin

Initial seeding density defines duration of the proliferative phase in the culture and therefore cells seeded at lower density would continue to divide for a longer period compared to cells seeded at a higher density until the cell population reaches confluency. Since TCblR expression is highest in actively dividing cells and down-regulated in resting cells, we tested cell lines at seeding densities ranging from 1000 – 10,000 cells/well with 2.5 nM mAb-Saporin concentration.

### Measuring functional receptor on cells

Functional receptor activity in whole cells was determined by incubating cells with ^57^CoB_12_ labeled TC. For suspension cultures, cells are collected by centrifugation, resuspended in DMEM and 0.5 to 1 × 10^6^ cells are suspended in 1 ml DMEM containing 10,000 CPM of radio-labeled TC at 4°C, or 37°C. An identical tube containing 10 mM EGTA is included to show blocking of the uptake. After 1 hour incubation, cells are pelleted by centrifugation at 2000 g for 5 min. washed once with buffer and counted for radioactivity. Adherent cells are seeded in 6 well culture plates, washed with DMEM and incubated with ^57^CoB_12_-TC in 1 ml DMEM for 1 hour. The cell layer is washed with HBS, incubated with 0.5 ml trypsin/ EDTA for 5min at 37°C, cells collected with solution and counted for radioactivity.

### Determination for mAb-Saporin concentration for inhibiting cell growth by 50% (IC50)

Since the toxic effect of mAb-Saporin was more pronounced in cell cultures seeded at lower density, the IC50 determinations were done with cells seeded at 2000 cells/well in 96 well plates with a mAb-Saporin concentration of 0.156 to 5 nM for 96 hours. The number of viable cells was determined by the MTS assay.

### Specificity of TCblR pathway for delivering the mAb- Saporin toxin

The specificity of the TCblR-mediated pathway for uptake and internalization of the mAb-Saporin complex was determined by adding recombinant soluble extracellular fragment of receptor to the culture medium. The soluble receptor would compete with the cell surface receptor for the antibody and this would reduce the Ab-toxin available for cellular uptake resulting in a decrease in percent inhibition. For these studies, K562 cells and both normal and stably transfected HEK293 cells to over express the receptor were used. Cells were seeded in 96 well plates at 2000 cells/well and the amount of mAb-Saporin-Ab used was equivalent to the IC50 concentration.

Specificity of mAb-Saporin for the TCblR receptor A 100 fold excess primary mAb or normal mouse IgG was added to the incubation medium containing a mAb-Saporin concentration of 2.5 nM. A decrease in the mAb-Saporin induced inhibition of cell growth should be observed when excess primary Ab without Saporin is present since the ratio of mAb-Saporin to unlabelled mAb should be lower and this increases the probability of unlabelled mAb binding to TCblR. The addition of normal mouse IgG should not result in a decrease in cell-kill since this cannot bind to TCblR and therefore would not compete with mAb-Saporin for binding to TCblR.

### Effect of mAb-Saporin on normal and cancer cells in culture

In order to determine any differential effect of the mAb-Saporin in targeting cancer cells in culture, various normal and cancer cell lines seeded at 1000, 2000 and 4000 cells/well were exposed to mAb-Saporin at an antibody concentration of 2.5 nM.

### Immunohistochemical staining of TCblR on cells

Cell lines cultured on gelatin-coated cover slips were fixed in 70% ethanol for 2 hrs at 4°C and washed in PBS. Human placental tissue was fixed in paraformaldehyde, embedded in Paraplast and 5 um sections were used for immunostaining. Rabbit polyclonal Ab to human TCblR (RPI) at 1:500 dilution was added for 1 hr, washed and incubated with biotin conjugated secondary Ab for 1 hr followed by ABC reagent (Vector Labs) for 30 min and color development with DAB (Vector Labs).

## 3. Results

**Figure 1:**
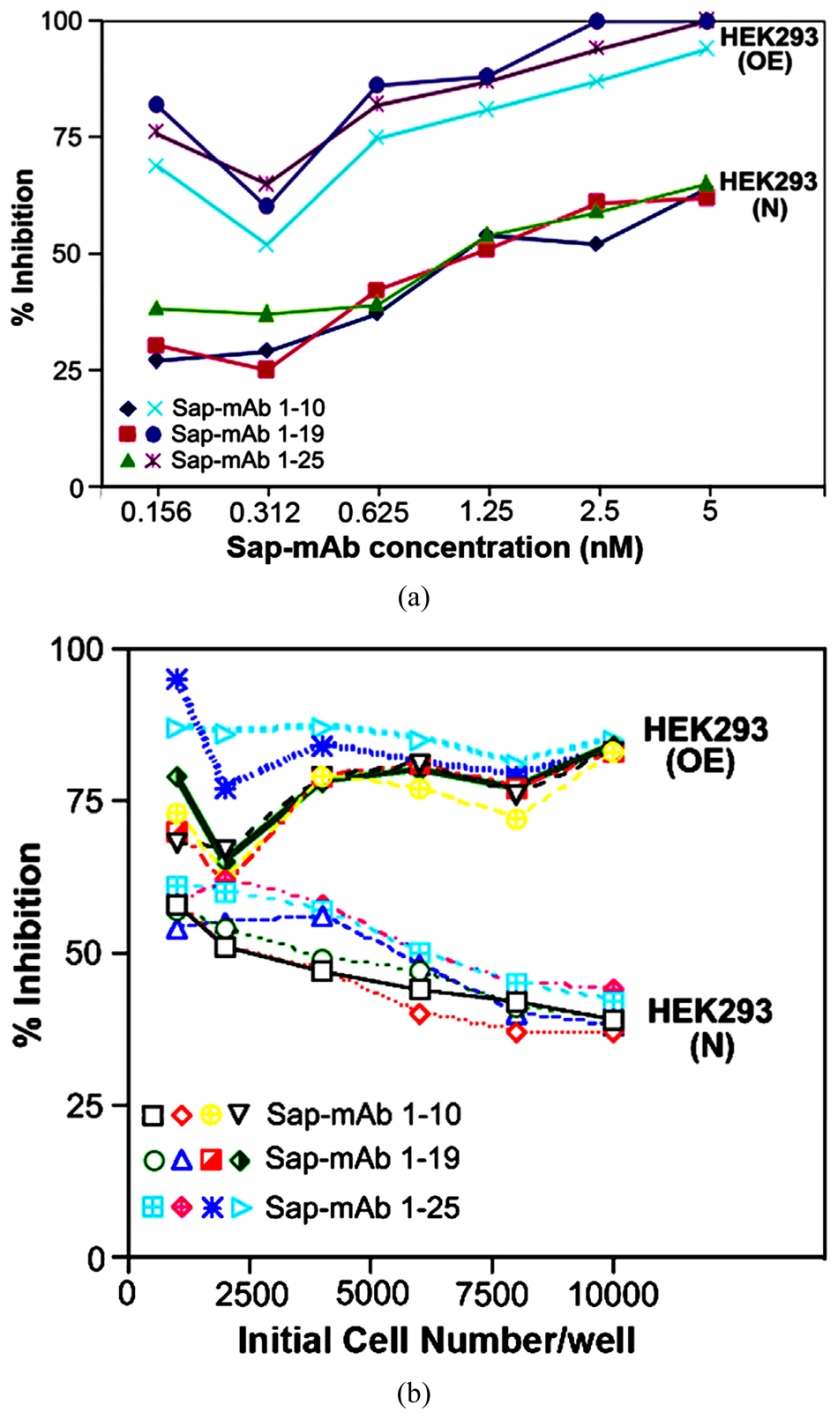
Effect of antibody concentration (a) and seeding cell density (b) on cell proliferation. Normal HEK 293 cells (N) and HEK 293 cells transfected to stably over express TCblR (OE) were tested for inhibition of cell proliferation during a 72 hour exposure to anti TCblRmAb-Saporin conjugate in culture. Results shown are mean of duplicate samples that varied <5%.

A number of adherent and suspension cell cultures were propagated in medium containing 0.156 to 5 nM mAb-Saporin. The effective concentration determined by percent decrease in viable cell number showed maximum effect at an antibody concentration of 2.5 to 5 nM. An example of this is shown in Figure 1(a) whereby normal HEK293 cells (293N) and those stably transfected to over express TCblR (293OE) showed a dose dependent effect with maximum inhibition of cell growth between 2.5 and 5 nM mAb-Saporin concentration. In addition, in 293N cells, inhibition of proliferation was influenced by initial seeding density which influences the rate and duration of proliferation and this effect was abrogated in 293OE cells which constitutively over express the receptor, independent of the cell cycle. In addition, 293OE cells were more severely affected compared to 293N cells due to sustained over expression of TCblR (Figure 1(b)). A mAb concentration of 2.5 nM was chosen for subsequent studies to ensure adequate extra cellular concentration of mAb-Saporin for all cell lines tested. Unconjugated Saporin at 10 to 100 fold molar excess had no effect on cell proliferation (data not shown).

**Figure 2:**
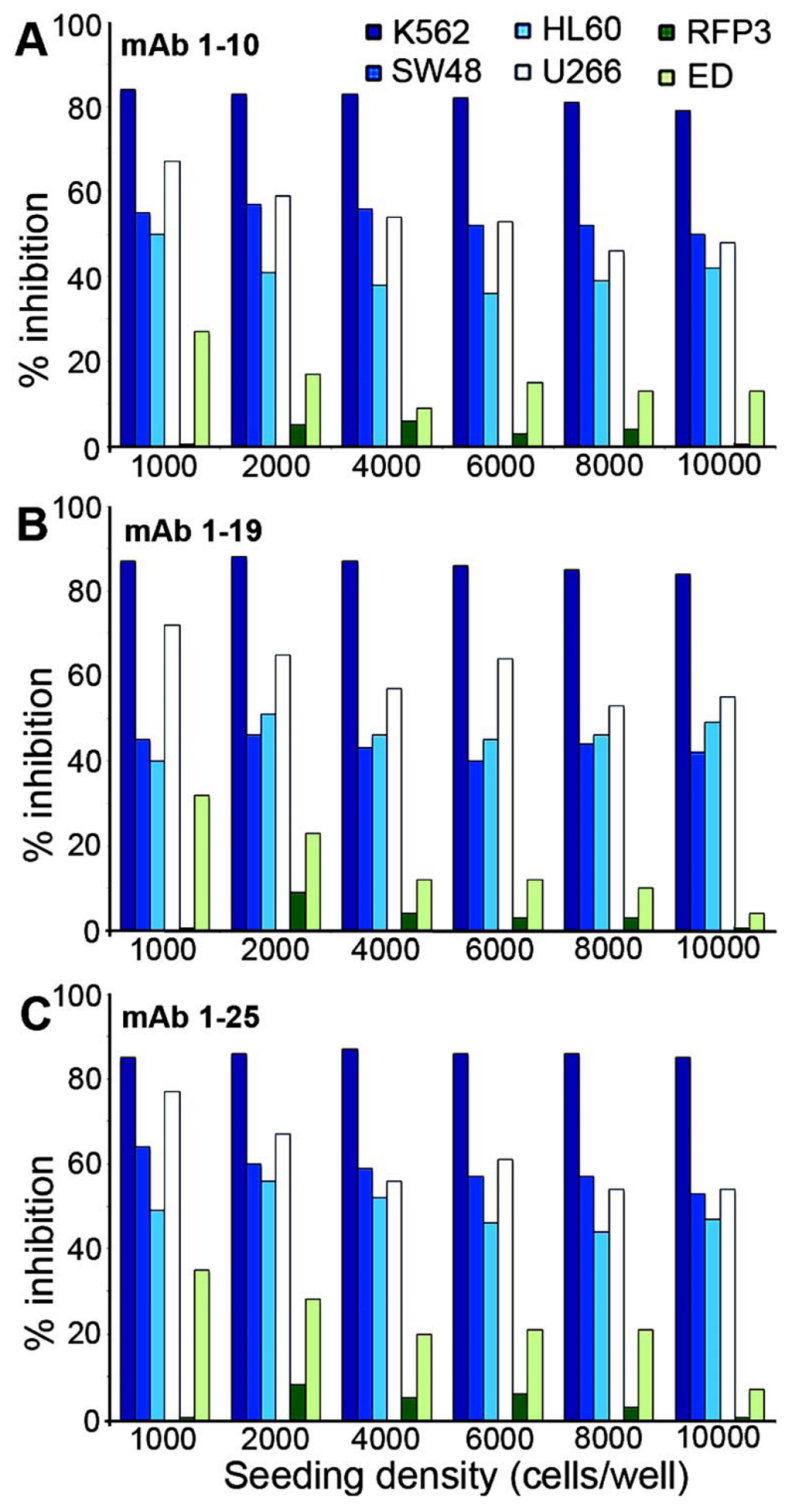
Effect of initial seeding density on proliferation of cells in the presence of antibody-Saporin conjugate. Normal primary cell lines and neoplastic cell lines were seeded at a density of 1000 – 10,000 cells per well in 96 well plates with 2.5 nM mAb-Saporin conjugate. Results shown are mean of duplicate samples that varied <5%.

Since initial seeding cell density is likely to affect the rate of proliferation and time to confluence, we tested a number of suspension and adherent cancer cell lines at initial seeding densities of 2000 – 10,000 cells per well in 96 well plates. The effect of mAb-Saporin was least influenced by the initial seeding density in both suspension and adherent tumor cells in culture, but the effect on cell proliferation was more pronounced in suspension cultures (Figure 2). A skin fibroblast cell line (RFP3) and a trophoblast line (ED) derived from human placenta (15) served as non-cancerous cell lines and were not affected or minimally affected (Figure 2).

**Figure 3:**
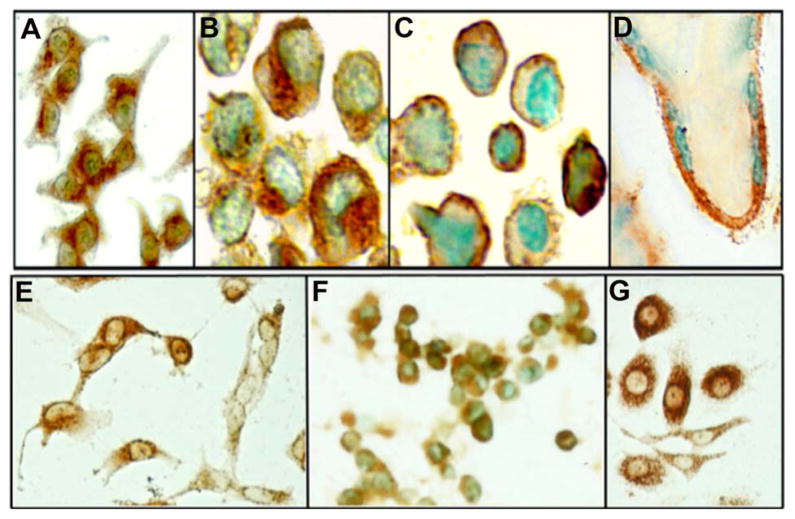
Immunohistochemical identification of TCblR expression in various human tissues. (A) KB epidermoid carcinoma; (B) HL60 acute promyelocytic leukemia; (C) U266 myeloma; (D) human placenta, E: U373 glioblastoma-astrocytoma; (F) HEK293 embryonic kidney; (G) PC3 prostate adenocarcinoma.

Since in normal cells, the receptor expression is cell cycle assocated and lower seeding density and higher receptor expression appear to have a greater inhibitory effect on cell proliferation, the lack of this effect in cancer cells was puzzling and therefore, we evaluated the expression of functional TCblR in a number of tumor and normal cell lines. Since TCblR expression appears to be cell cycle dependent, we determined cell surface receptor expression by TC-Cbl binding at various time points during a 96 hour culture period. As seen in Table 1, TCblR expression varied considerably among the various tumor lines. In some cell lines TCblR expression peaked between 8 – 48 h and was followed by a substantial decrease in TC-Cbl binding by 96h, a profile observed for most normal cell lines. In some tumor lines, TC-Cbl was substantially up regulated initially and did not decrease to the level seen in normal cells. On the other hand, some tumor lines expressed moderate level of TCblR throughout the culture period without the peak and decrease seen in normal cells. Immunohistochemical visualization of the receptor showed specific membrane staining of TCblR (Figure 3). While the level of TCblR expression is too low to detect in most normal cell lines such as skin fibroblasts, the expression is adequate to visualize in human placenta and most tumor cell lines.

**Figure 4:**
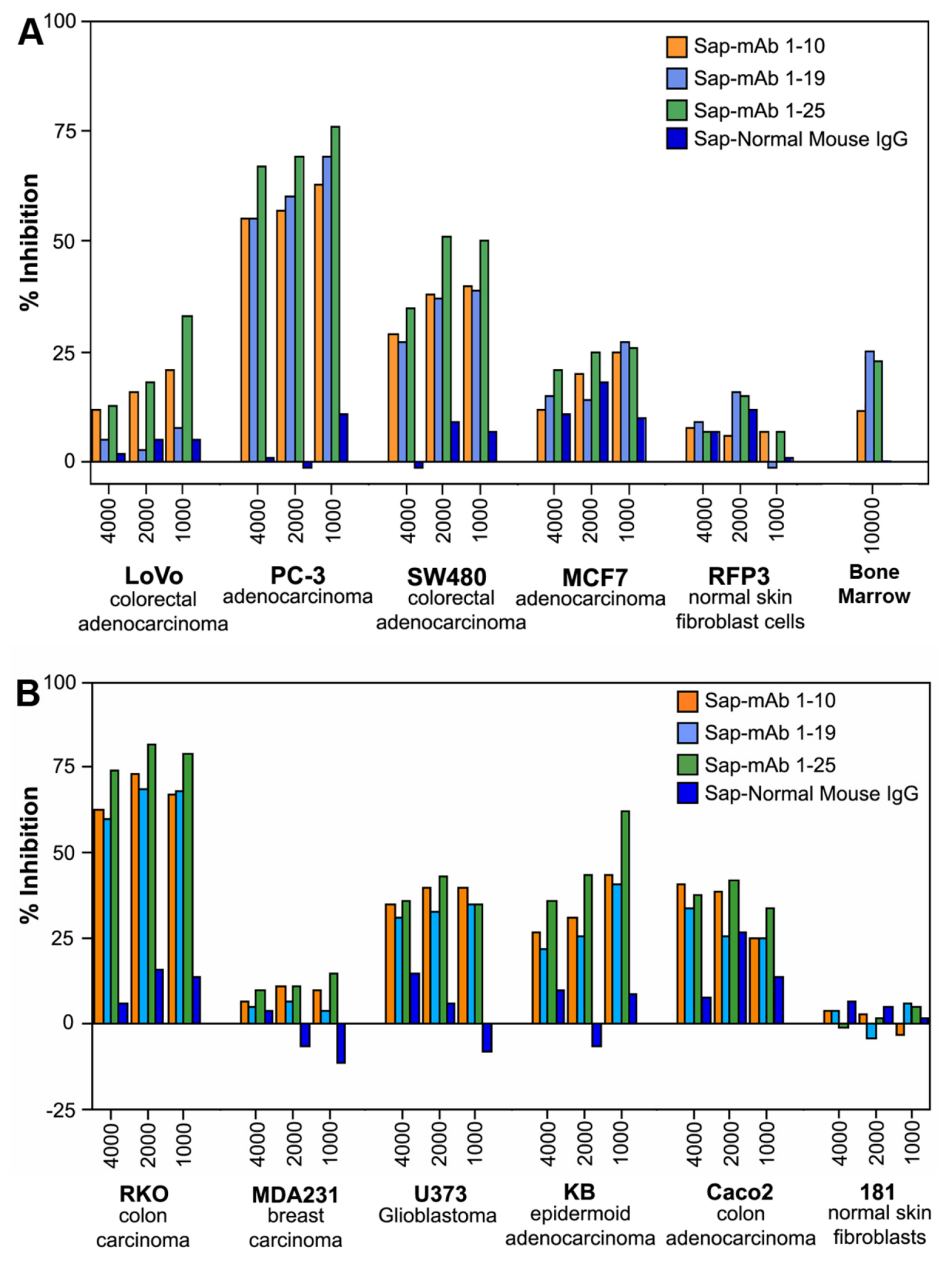
Inihibition of cell proliferation in the presence of mAb-Saporin conjugates. Data shows various tumor cell lines with differences in susceptibility to the toxin. Results shown are mean of duplicate samples that varied <5%.

**Figure 5:**
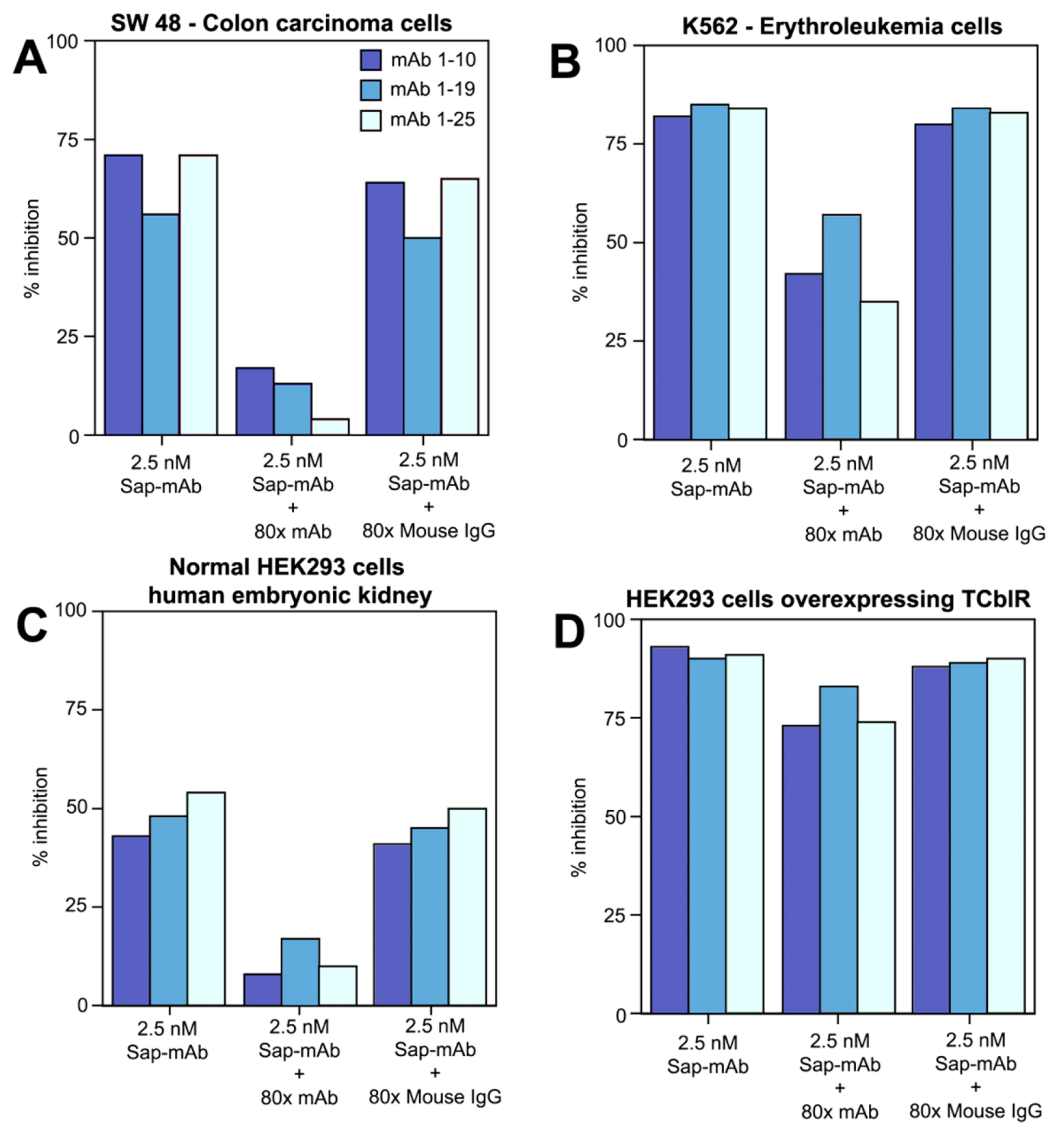
The specificity of mAb-Saporin conjugate for TCblR. Figure shows excess unconjugated specific mAb decreases the inhibitory effect of the mAb-Saporin conjugate while normal mouse IgG does not (A) & (C). This effect is less pronounced in suspension cultures (B) and in cells over expressing TCblR (D). Results shown are mean of duplicate samples that varied <5%.

**Figure 6:**
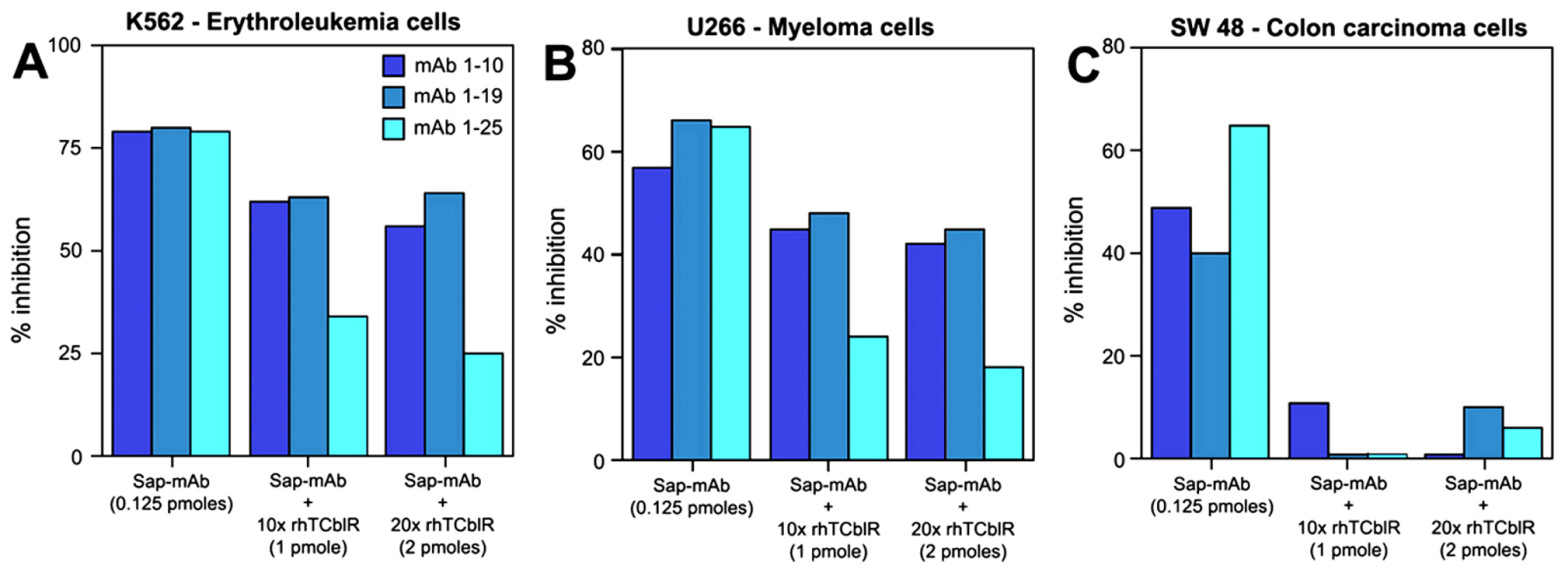
The specificity of targeting TCblR by Mab-Saporin conjugate. The recombinant extracellular domain of TCblR added to culture decreases the inhibitory effect of the mAb-Saporin conjugate in culture. Results shown are mean of duplicate samples that varied <5%.

Because of the wide range of doubling time and the 8 – 48 h period for peak receptor expression in various tumor lines, the cytotoxic effect of the mAb-saporin was determined at three different initial cell densities of 1000, 2000 and 4000 cells and we evaluated three monoclonals with specificity for different regions of the protein (10). As shown in Figure 4, typically, lower seeding density showed higher inhibition of cell growth and the effect varied with different cell lines. Table 1 provides the IC50 inhibitory dose for the three mAb-Saporin conjugates on cell proliferation. All three antibodies were effective as carriers of Saporin and the average inhibition observed for a specific cell line was similar for all three antibodies. However, there were major differences in susceptibility of different cell lines to the mAb-Saporin conjugate. On average, most of the suspension cell lines showed higher inhibition of cell proliferation with K562 and RPMI18266 showing the most inhibition. Among the adherent cell lines also, the effect varied with RKO, SW48, HeLa and PC3 showing the most inhibition and LoVo. Hep G2, Hep 3B, MCF7, MDA-MB-231 and MIA PaCa-2 cell lines, showing least inhibition. Inhibition of cell proliferation was more pronounced in suspension cultures than in adherent cultures at all concentrations of the Saporin-antibody and cell density tested. In cell lines with 50% or less inhibition of growth, increasing the antibody concentration did not increase the inhibition. Based on the above data, the IC 50 for most tumor cell lines appears to be below 100 pM (Table 1). The specificity of Saporin conjugated antibodies was evident from a decrease in inhibition with the addition of excess unconjugated specific antibody and lack of any effect with excess normal mouse IgG (Figure 5). The specificity of receptor targeting by the antibodies was further confirmed by adding soluble recombinant TCblR to the incubation, which reduced the effect of the Saporin conjugated antibodies (Figure 6).

## 4. Discussion

Our previous study described the utility of monoclonal antibodies to the extracellular domain of TCblR as effective carriers of Saporin conjugated secondary antibody into cells [11]. Encouraged by this preliminary observation, we have extended the approach to evaluate the efficacy of Saporin directly conjugated to primary antibodies. Two of the three representative antibodies (TCblRKB1-10 and TCblRKB 1–19) selected were binding antibodies, in that binding of these antibodies to their epitopes on TCblR did not prevent the binding and internalization of the physiologic ligand, TC-Cbl. The third antibody (TCblRKB1-25) was a blocking antibody since binding of this antibody to TCblR, prevented the binding and cellular uptake of TC-Cbl. The epitope specificity of these antibodies and properties are described elsewhere [10]. All three mAb-Saporin conjugates are fully functional in recognizing the target antigen with high affinity, an important requirement for mAbs to be used as carriers of drugs and toxins. Even though a marginal increase in internalization of mAb 1 – 10 and 1 – 19 is observed with TC-Cbl in the culture medium, the binding of the native ligand is not a pre requisite for antibody binding and internalization. Since in the absence of TC-Cbl, the apo receptor remains on the plasma membrane, TC-Cbl binding appears to trigger the necessary response for internalization. The effective binding and internalization of all antibodies, including the ligand blocking antibody, mAb 1 – 25 is strong evidence that antibody binding triggers a response similar to that of ligand TC-Cbl binding. Highly potent toxins such as ricin, cholera toxin, gelonin and saporin, drugs or radionuclides are very effective in destroying cancer cells if a toxic dose can be delivered specifically to these cells [16,17]. This strategy requires a tumor specific carrier to transport the toxin across the plasma membrane into cells since these molecules cannot cross the cell membrane by either specific or nonspecific transport mechanisms. An ideal target protein would be a receptor or cell surface protein that is expressed and internalized only in cancer cells. However, such proteins are scarce and not easy to identify. Many proteins and receptors are expressed in all cell types and some of these are cell cycle associated or expressed only in actively dividing cells. Such proteins can be carriers for drugs and toxins and may provide some degree of enhanced targeting to cancer cells. However selective targeting to cancer cells and lack of toxicity to the normal cell population will depend not only on the differential expression but also on the density of the target protein in the two cell types. For example, a protein with relatively high expression such as the transferrin receptor [18], even though differentially expressed in cancer and normal cells, would not be suitable for delivering a toxin because the normal cells would internalize sufficient toxin to kill the cell. The ideal target protein is one with fairly low expression in normal cells and cannot internalize toxic amounts of drugs but is adequately over expressed in cancer cells to internalize cytotoxic amounts of the drug. The TCblR is one such protein whose expression is sufficiently low to render any toxin internalized in normal cells to be ineffective and is adequately over expressed in some cancers to internalize sufficient toxin to kill the cell. In addition, the cell cycle associated expression of this protein makes highly proliferative cancer cells with sustained expression, an ideal target for this approach. Blocking Cbl uptake into cells with monoclonal antibody to TC can deplete cells of Cbl and ultimately inhibit DNA synthesis leading to inhibition of cell replication [19,20]. A specific antibody to TCblR that blocks the binding of TC-Cbl could also have the same effect [10]. This approach, even though less toxic, is likely to be slow and many cancers require a faster effect to destroy the malignant tissue before it metastasizes. Inhibiting CD320 expression with siRNA also affects proliferation by depleting intracellular Cbl [21]. The use of potent drugs or toxins conjugated to antibody that can deliver the payload to its target antigen is a highly effective strategy that can provide the specificity and speed of action demanded in cancer therapy. The present data on the use of mAb-Saporin conjugate to target TCblR appears to be specific for certain cancers and provides proof of concept for utilizing this receptor for targeted delivery of drugs and toxins and awaits confirmation of *in vivo* targeting efficacy of this pathway.

## Figures and Tables

**Table 1:** TCblR expression profile and concentration of mAb-Saporin required for inhibiting cell proliferation.

				Cbl-TC binding					IC50/2000 cells (nM)		

Cell Line	Source	Cell line discription	Doubling	pg/10^6^ cells					1 – 10 saporin	1 – 19 saporin	1 – 25 saporin

			Time hr	8 – 18 hr	24 hr	48 hr	72 hr	96 hr			
K-562*	ATCC CCL-243	chronic myelogenous leukemia (CML)	24 – 40	10	19	10	7	3	<0.05	<0.05	<0.05
HL-60	ATCC CCL-240	acute promyelocytic leukemia	24	4	6	5	4	3	11	10	10
Jurkat	ATCC TIB-152	acute T-cell leukemia	25 – 35	20	21	15	6	4	2.4	2.5	2.08
U266B1 [U266]*	ATCC TIB-196	myeloma plasmacytoma	55	10	10	7	7	8	0.8	<0.05	<0.05
RPMI8226*	ATCC CCL-155	plasmacytoma; myeloma, B lymphocyte	60 – 70	6	15	15	8	7	1.92	1.62	1.54
NCI-H929 [H929]*	ATCC CRL-9068	Plasmacytoma, Myeloma	70	20	24	24	33	15	1.89	1.42	1.49
U4937 (U937)*	ATCC CRL-1593.2	monocytic cell, histocytic lymphoma	20 – 48	9	9	10	3	3	1.87	1.58	1.64
SW48 [SW-48]*	ATCC CCL-231	colorectal adenocarcinoma, epithelial	35	20	37	18	10	7	<0.05	1.7	<0.05
SW480 [SW-480]	ATCC CCL-228	colorectal adenocarcinoma, epithelial	25 – 48	19	15	12	7	10	3.29	3.38	2.45
RKO*	ATCC CRL-2577	carcinoma colon, epithelial	14	26	27	11	8	8	1.71	1.81	1.52
LoVo	ATCC CCL-229	colorectal adenocarcinoma, epithelial	36 – 48	9	7	10	12	14	7.81	41.7	6.94
Caco-2	ATCC HTB-37	colorectal adenocarcinoma	24 – 62	6	8	7	5	4	3.21	4.81	2.98
Hep G2	ATCC HB - 8065	hepatocellular carcinoma (epithelial)	50 – 60	17	14	9	3	3	10.4	10.4	4.81
Hep 3B	ATCC HB - 8064	hepatocellular carcinoma (epithelial)	40 – 50	11	11	10	9	6	5.95	6.25	4.03
KB	ATCC CCL-17	HeLa Derivatives	30 – 40	38	16	15	11	5	4.03	4.81	2.84
MDA-MB-231	ATCC HTB-26	adenocarcinoma, mammary gland; breast	50 – 60	33	45	10	6	8	11.3	17.9	11.3
MCF7	ATCC HTB-22	adenocarcinoma, mammary gland; breast	50	12	28	21	10	7	6.25	8.93	5
HeLa	ATCC CCL-2	cervix, adenocarcinoma (epithelial)	48	13	12	13	9	9	2.11	2.55	1.84
A431NS	ATCC CRL-1555	epidermal carcinoma: skin	80 – 100	3	3	4	4	3	6.58	9.62	4.63
MIA PaCa-2	ATCC CRL-1420	Pancreatic carcinoma	40	22	11	11	7	7	5	10.4	5.2
PC-3*	ATCC CRL-1435	prostate adenocarcinoma	50	16	16	18	10	10	2.19	2.08	1.81
U-373 MG	ATCC HTB-17	identities in question	24 – 48	19	8	8	6	7	3.1	3.79	2.91
HEK-293 Normal	ATCC CRL-1573	human embryonic kidney cells	24 – 30	9	9	9	5	6	4.3	4.1	3.7
EC304 (ECV 304)	ATCC CRL-1998	T24 (human bladder cell) derivative	48	13	16	6	5	5	2.91	3.29	2.5
ED	Quadros lab	embryonic cells from human placenta	48	8	13	2	2	2	12	14	12
RFP3	Quadros lab	human skin fibroblast	18 – 24	3	13	2	2	2	30	24	26
2M-125C	Lonza 2M-125C	human bone marrow MNC	Not tested						5	4.8	4.8

Cells for TCblR expression were seeded in 6 well plates at a density of 0.2 × 10^6^ cells/well and Cbl-TC binding was determined by incubating with [^57^Co] Cbl-TC (10,000 cpm) for 1hr at 37 °C in 1 ml medium. The effect of mAb-Saporin on cells was determined at 72 hr following seeding of 2000 cells/well in a 96 well plate with 0.156 – 5 nM mAb-Saporin and IC50 (concentration for inhibition cell proliferation by 50%) was calculated from the slope of the growth curve.

* Asterisk indicates cells most susceptible to mAb-Saporin.
